# Role of Cholecystectomy on Serum Lipid Profile in Patients With Gallstone Disease at Tertiary Care

**DOI:** 10.7759/cureus.82691

**Published:** 2025-04-21

**Authors:** Ganugapanta Prem Sai Reddy, V Ramalakshmi, Alexander Mecheri Antony, T Raghupathy, Ganesh Guru

**Affiliations:** 1 General Surgery, Sree Balaji Medical College and Hospital, Chennai, IND

**Keywords:** cholecystectomy, cholesterol, cholesterol stones, gallstones, hdl, pigment stones, serum lipid profile

## Abstract

Background: Metabolic syndrome is a known risk factor for gallstone formation. Elevated cholesterol concentration in the bile contributes to the formation of cholesterol stones. Following cholecystectomy, lipid profile abnormalities tend to normalize.

Objectives: This study aims to evaluate serum lipid profile parameters before and after cholecystectomy and to assess their relationship with the type of gallstones.

Materials and methods: The present study was a prospective observational study conducted in the Department of General Surgery at Sree Balaji Medical College and Hospital, Chennai, Tamil Nadu, India, between August 2023 and September 2024. Ethical clearance was obtained from the institutional ethics committee. The study included patients diagnosed with with gallstones who consulted the Department of General Surgery and underwent either open or laparoscopic cholecystectomy. Paired samples t-tests were used to compare lipid profile values before and after cholecystectomy.

Results: Among the participants, 49 (57.6%) were aged 41-60 years, and 22 (25.9%) were aged 18-40 years. Sixty-two (72.9%) were female patients, and 59 (69.4%) resided in urban areas. All lipid profile parameters decreased significantly following cholecystectomy, except for high-density lipoprotein (HDL) levels, which showed a significant increase. The mean serum cholesterol and low-density lipoprotein (LDL) levels were lower in patients with pigment stones compared to those with other types of stones.

Conclusion: Cholecystectomy in patients with gallstones was found to normalize deranged lipid profiles. Significant reductions were observed in total cholesterol, LDL, triglycerides, and very LDL (VLDL) levels following the intervention. HDL levels were also increased significantly following cholecystectomy.

## Introduction

The gallbladder is a pear-shaped organ within the biliary system responsible for storing bile secreted by hepatocytes. The biliary system begins at the segmental ducts, each of which may drain one or more hepatic segments. The ducts merge to form the right and left hepatic ducts. The extrahepatic biliary system comprises the right and left hepatic ducts, the common hepatic duct, and the gallbladder [1]. Gallstones can form in either the gallbladder or the biliary tract, typically due to high concentrations of cholesterol or bile [2]. Stones primarily composed of cholesterol are termed cholesterol stones, while those formed from calcium bilirubinate are classified as pigment stones. Mixed stones contain 50% cholesterol or more, with the remainder consisting primarily of calcium bilirubinate [3].

Cholesterol stones result from a disruption in bile cholesterol homeostasis. Excessive cholesterol secretion or reduced secretion of bile salts or phospholipids can lead to bile supersaturation with cholesterol. When high cholesterol concentrations coexist with specific proportions of bile salts and phospholipids, crystallization may occur [4]. Pigment stones arise from abnormalities in bilirubin metabolism and may be black or brown in color. Black stones usually form from calcium bilirubinate, whereas brown stones develop from calcium salts of unconjugated bilirubin [5-7].

Metabolic syndrome has been identified as a risk factor for gallstones, with high cholesterol concentrations in bile contributing to the formation of cholesterol stones. Studies have shown a higher prevalence of dyslipidemia in individuals with gallstones compared to those without [8]. Additionally, following cholecystectomy, lipid profile abnormalities tend to normalize. In the long term, this may lead to a reduced incidence of cardiovascular disease in those suffering from gallstone disease [9,10].

The present study aimed to assess serum lipid profile parameters before and after cholecystectomy and to examine the relationship between serum lipid profiles and gallstone type. Few previous studies with similar objectives have been conducted in this population. This study provides valuable insight into the effect of cholecystectomy on lipid profiles in individuals with gallstone disease.

## Materials and methods

The present study was a prospective observational study conducted in the Department of General Surgery, Sree Balaji Medical College and Hospital, Chennai, Tamil Nadu, India, between August 2023 and September 2024. Ethical clearance was obtained from the institutional ethics committee. Study participants included patients diagnosed with gallstones who consulted the Department of General Surgery and underwent either open or laparoscopic cholecystectomy. Inclusion criteria were patients with symptomatic cholelithiasis undergoing open or laparoscopic cholecystectomy. Exclusion criteria included patients with known hyperlipidemia (with or without medication), renal failure, pancreatitis, cardiac failure, cardiac conditions requiring lipid-lowering therapy, hypothyroidism, pregnancy, and obstructive jaundice.

The sample size for the study was calculated using the following formula: n = Z^2^ * P(1-P)/d^2^, where z is the statistical constant corresponding to the level of confidence (1.96), P is the estimated prevalence (5%), and d is the allowable error (0.05). The calculated sample size was 75. After adjusting for a 10% non-response rate, the final sample size was set at 85. Data were collected using a pre-tested, semi-structured proforma. All patients who met the inclusion criteria and did not meet any exclusion criteria were approached for participation in the study.

The purpose of the study was explained to all participants in advance, and informed written consent was obtained. Data collected included participants' age, sex (male or female), and place of residence (urban and rural, indicating residence in a city/town or a village, respectively). Behavioral factors such as smoking and alcohol use were also recorded. Female participants were asked about their history of oral contraceptive use. documented as either "yes" or "no."

A history of comorbidities was obtained from the participants. Present symptoms at the time of admission, such as pain, nausea, and vomiting, were recorded. Clinical examination findings were documented, including the presence of jaundice (indicated by yellowish discoloration of the sclera), abdominal tenderness, and Murphy’s sign.

Participants' height was measured to the nearest 0.1 cm using a measuring tape, and weight was measured to the nearest 0.1 kg using a digital weighing scale. Body mass index (BMI) was calculated using the standard formula: weight in kilograms divided by the square of the height in meters (kg/m^2^). Preoperative serum lipid profile values were also obtained for all participants.

Following surgery, either open or laparoscopic cholecystectomy, the type and number of gallstones were recorded. For comparability with existing literature, the traditional classification of gallstones into cholesterol, pigment, and mixed types was used. A postoperative lipid profile was obtained at three months during the follow-up period and compared with the preoperative lipid profile.

## Results

Among the participants, 49 (57.6%) were aged 41-60 years and 22 (25.9%) were 18-40 years. Sixty-two (72.9%) were female patients, and 59 (69.4%) resided in urban areas. Twelve participants (14.1%) were smokers, and 15 (17.6%) reported alcohol use. Among the female participants, 24 (38.7%) had a history of oral contraceptive use. In terms of comorbidities, 22 (25.9%) had diabetes, and 31 (36.5%) had hypertension. Regarding nutritional status, 33 (38.8%) were of normal weight, and 27(31.8%) were classified as overweight (Table 1).

**Table 1:** Sociodemographic characteristics, risk factors, and comorbidities of participants.

Variables		Frequency (n = 85)	Percentage
Age group (in years)	18-40	22	25.9
	41-60	49	57.6
	>60	14	16.5
Sex	Male	23	27.1
	Female	62	72.9
Place of residence	Urban	59	69.4
	Rural	26	30.6
Smoking	Present	12	14.1
	Absent	73	85.9
Alcoholic	Yes	15	17.6
	No	70	82.4
History of oral contraceptive use	Present	24	38.7
	Absent	38	61.3
Comorbidities	Diabetes mellitus	22	25.9
	Hypertension	31	36.5
	Bronchial asthma	8	9.4
	Hypothyroid	12	14.1
BMI (kg/m^2^)	<18	7	8.2
	18-25	33	38.8
	​26-30​	27	31.8
	>30	18	21.2

In the present study, 42 (49.4%) presented with right hypochondriac pain, and 26 (30.6%) reported epigastric pain at the time of admission. Both nausea and vomiting were observed in 43 (50.6%), while 26 (30.6%) experienced vomiting alone. Jaundice was noted in 18 (21.2%), abdominal tenderness in 57 (67.1%), and a positive Murphy’s sign in 38 (44.7%). Regarding the type of gallstones, 48 (56.5%) had pigment stones, 22 (25.9%) had mixed stones, and 15 (17.6%) had cholesterol stones. Single stones were found in 37 (43.5%), while 48 (56.5%) had multiple stones. Open cholecystectomy was performed in 37 participants (43.5%), and laparoscopic cholecystectomy in 48 (56.5%) (Table 2).

**Table 2:** Distribution of symptoms, signs, and type of gallstones.

	Symptoms		Frequency (n=85)	Percentage (%)
Symptoms	Abdominal pain	Epigastric pain	26	30.6
		Right hypochondriac pain	42	49.4
	Nausea		7	8.2
	Vomiting		26	30.6
	Nausea and vomiting		43	50.6
Signs	Jaundice		18	21.2
	Abdominal tenderness		57	67.1
	Murphy’s sign		38	44.7
Type of stones	Cholesterol stones		15	17.6
	Pigment stones		48	56.5
	Mixed stones		22	25.9
Number of stones	Single		37	43.5
	Multiple		48	56.5
Type of surgery	Open cholecystectomy		37	43.5
	Laparoscopic cholecystectomy		48	56.5

The mean total serum cholesterol level decreased from 211.55 ± 48.92 mg/dL before cholecystectomy to 189.41 ± 38.67 mg/dL after the procedure. The mean serum triglyceride level reduced from 178.18 ± 51.03 mg/dL to 162.88 ± 45.09 mg/dL. The mean serum HDL level increased from 38.45 ± 6.39 mg/dL preoperatively to 41.53 ± 5.89 mg/dL postoperatively. In contrast, the mean serum LDL level decreased from 137.81 ± 38.47 mg/dL to 122.84 ± 29.67 mg/dL. Similarly, the mean VLDL level decreased from 35.73 ± 8.31 mg/dL to 31.14 ± 13.53 mg/dL. All lipid profile parameters decreased significantly following cholecystectomy, except for HDL levels, which showed a significant increase (Table 3). 

**Table 3:** Comparison of serum lipid levels before and after cholecystectomy. HDL: high-density lipoprotein, LDL: low-density lipoprotein, VLDL: very low-density lipoprotein.

Variable	Cholecystectomy		T-value	p-value
	Before	After		
Total serum cholesterol levels	211.55 ± 48.92	189.41 ± 38.67	3.27	0.001
Serum triglyceride levels	178.18 ± 51.03	162.88 ± 45.09	2.07	0.039
Serum HDL levels	38.45 ± 6.39	41.53 ± 5.89	3.26	0.001
Serum LDL levels	137.81 ± 38.47	122.84 ± 29.67	2.84	0.005
Serum VLDL levels	35.73 ± 8.31	31.14 ± 13.53	2.66	0.008

The mean serum cholesterol level was 221.31 ± 42.81 mg/dL among patients with cholesterol stones, 218.41 ± 39.73 mg/dL for those with pigment, and 181.38 ± 31.54 mg/dL for those mixed stones. The mean total cholesterol level was distinct between the various stone categories with p < 0.05. The mean level was lower for pigment stones than the rest of the stones. The mean serum LDL level was 139.05 ± 32.97 mg/dL in the cholesterol stone group, 105.11 ± 16.27 mg/dL in the pigment stone group, and 131.47 ± 15.82 mg/dL in the mixed stone group. The mean serum LDL level was distinct between the various stone categories with a p < 0.05. The mean serum LDL level was lower for pigment stones than for the other types of stones. The mean triglyceride, HDL, and VLDL levels were similar across different type of stones (p > 0.05) (Table 4).

**Table 4:** Mean lipid profiles across different types of gallstones before intervention. HDL: high-density lipoprotein, LDL: low-density lipoprotein, VLDL: very low-density lipoprotein.

Parameters	Cholesterol stones (n = 15)	Pigment stones (n = 48)	Mixed stones (n = 22)	F-value	p-value
Serum total cholesterol (mg/dL)	221.31 ± 42.81	181.38 ± 31.54	218.41 ± 39.73	11.88	0.001
Serum triglyceride (mg/dL)	179.21 ± 49.81	165.48 ± 58.91	176.54 ± 47.54	0.52	0.593
Serum HDL (mg/dL)	36.73 ± 6.08	43.13 ± 14.71	39.45 ± 9.37	1.78	0.175
Serum LDL (mg/dL)	139.05 ± 32.97	105.11 ± 16.27	131.47 ± 15.82	23.13	0.001
Serum VLDL (mg/dL)	36.09 ± 6.72	32.81 ± 18.43	36.87 ± 8.11	0.40	0.666

## Discussion

Gallstones are formed either due to elevated cholesterol or bilirubin levels present in the bile. A higher probability of occurrence of gallstones was linked to advanced age and female sex. It was also found to be associated with the presence of metabolic syndrome. The pathogenesis behind the formation of gallstones includes a high concentration of cholesterol in bile, cholesterol crystal formation, poor gall bladder emptying alongside stasis of its contents, and hypermotility of the intestine. The presence of disruption in lipid profile among those with gallstones is well-documented, and studies have shown a significant change in lipid profiles following cholecystectomy. The present study was carried out to assess the impact of cholecystectomy on serum lipid profile in patients with gallstone disease [11].

About half of the participants were aged 41-60 years and a quarter were in the age group 18-40 years. The ratio of female-to-male participants was 2:1. Farhat et al. reported women to be more prone to cholelithiasis than men. In the present study, the proportion of female participants was higher than that of male participants [12].

Before cholecystectomy, the mean serum total cholesterol level was 211.55 ± 48.92 mg/dL, mean serum triglyceride level was 178.13 ± 51.03 mg/dL, mean serum HDL level was 38.45 ± 6.39 mg/dL, mean serum LDL level was 137.81 ± 38.47 mg/dL, and mean serum VLDL level was 35.73 ± 8.31 mg/dL. Olokoba et al. reported higher serum cholesterol and triglyceride levels among those with gallstones than those without [13]. Malik et al. reported that among those with gallstone disease, about 80% and 71.4% of women and men, respectively, had abnormal lipid profiles [14]. Sabanathan et al. conducted a study to quantify dyslipidemia among patients with cholelithiasis and documented the presence of hypercholesterolemia, hypertriglyceridemia, and raised LDL levels among the participants [15]. The study also found that more than 70% of participants of both sexes had dyslipidemia [15]. A cross-sectional study by Hayat et al. reported that among those with gallstones, triglyceride levels were significantly higher, and HDL levels were significantly lower than those without gallstones [16]. Farhat et al. reported a disrupted serum lipid profile as a risk factor for the development of cholelithiasis [12].

The mean total cholesterol, mean triglyceride, mean LDL, and mean VLDL levels decreased significantly following cholecystectomy, and the mean HDL levels increased significantly following cholecystectomy in the present study. Jindal et al. reported a similar change in lipid profile following cholecystectomy [11]. The study reported a significant decline in the total cholesterol, triglyceride, and LDL levels following surgery. The study added that the mean HDL levels also significantly increased among the participants. These results were similar to that of the present study [11]. Another study from Uttar Pradesh, India, carried out by Kumar et al. reported similar changes in the serum lipid levels following cholecystectomy, a significant increase in HDL levels, and a significant reduction in the other lipid parameters during their follow-up exams on the 3rd, 7th, and 30th days [17].

Gill and Gupta reported that both the total cholesterol and triglyceride levels declined significantly following cholecystectomy, while no change occurred in the LDL and VLDL levels [18]. Their study also reported a significant rise in serum HDL levels following cholecystectomy [18]. Malik et al. reported that following cholecystectomy, there were significant decreases in the concentrations of triglycerides, total cholesterol, and LDL values in a study that followed participants until six months post-surgery [14].

A study by Osman et al. conducted among participants with gallstones who were symptomatic and had uncomplicated diseases reported that following cholecystectomy, the serum LDL values considerably reduced, as did the mean total cholesterol levels [19]. Alkataan et al. reported a significant reduction in mean total cholesterol levels among those with gallstones following cholecystectomy [20]. Ikram et al. conducted a similar study and documented a similar change in serum lipid profiles during the post-cholecystectomy phase among the participants. The study reported a significant reduction in serum total cholesterol during the phase, along with LDL and triglyceride levels. The study also added a significant increase in mean HDL levels during the phase [21].

The mean serum cholesterol and LDL levels were significantly higher among those with cholesterol or mixed stones than pigment stones or other stone types. The mean triglyceride, HDL, and VLDL levels were similar across different types of stones. Following cholecystectomy, the deranged lipid profile reverted toward normal values. There has been a significant reduction in the levels of total cholesterol, LDL, VLDL, and triglyceride values, while HDL levels increased significantly.

## Conclusions

Cholecystectomy in patients with gallstones was found to normalize the deranged lipid profile. Significant reductions were observed in total cholesterol, LDL, triglycerides, and VLDL levels following the intervention, while HDL levels showed a significant increase.
